# Improvement of the Self-Controlled Hyperthermia Applications by Varying Gadolinium Doping in Lanthanum Strontium Manganite Nanoparticles

**DOI:** 10.3390/molecules28237860

**Published:** 2023-11-30

**Authors:** Ashfaq Ahmad, Hassan Akbar, Imran Zada, Faiza Anjum, Amir Muhammad Afzal, Subhan Javed, Muhammad Muneeb, Asghar Ali, Jeong Ryeol Choi

**Affiliations:** 1 School of Material Science and Engineering, Shanghai Jiaotong University, Shanghai 200240, China; ashfaq@sjtu.edu.cn (A.A.);; 2 College of Environmental Science and Engineering, North China Electric Power University, 2 Beinong Road, Beijing 102206, China; 120214200011@ncepu.edu.cn; 3 Department of Physics, Abbottabad University of Science and Technology (AUST), Abbottabad 22010, Pakistan; 4 Pakistan Department of Physics, The University of Lahore, 1-Km, Defense Road, Lahore 54000, Pakistan; 5 Pakistan Department of Physics, Riphah International University, Lahore 54000, Pakistan; 6 School of Electronic Engineering, Kyonggi University, Yeongtong-gu, Suwon 16227, Gyeonggi-do, Republic of Korea

**Keywords:** magnetic nanoparticles, silica coating, gadolinium doping, Curie temperature, self-controlled magnetic hyperthermia, specific absorption rate

## Abstract

In this study, silica-encapsulated gadolinium was doped in lanthanum strontium manganite nanoparticles (NPs) with different concentrations using the citrate–gel auto-combustion method. We focused on tuning the Curie temperature and enhancing the specific absorption rate (SAR) of silica-coated gadolinium-doped lanthanum strontium manganite NPs to make them suitable for self-controlled magnetic hyperthermia. The samples were characterized by using transmission electron microscopy (TEM), X-ray diffraction, Fourier transform infrared spectroscopy (FTIR), and magnetic measurements to examine the structural, optical, and magnetic properties of the manganite NPs. While our results exhibit a successful doping of gadolinium in lanthanum strontium manganite NPs, we further prepared magnetic core NPs with sizes between 20 and 50 nm. The Curie temperature of the NPs declined with increasing gadolinium doping, making them promising materials for hyperthermia applications. The Curie temperature was measured using the magnetization (M-T) curve. Magnetic heating was carried out in an external applied AC magnetic field. Our present work proved the availability of regulating the Curie temperature of gadolinium-doped lanthanum strontium manganite NPs, which makes them promising candidates for self-controlled magnetic hyperthermia applications.

## 1. Introduction

Nanocrystalline magnetic nanoparticles (MNPs) are promising materials which have been broadly considered in the last few decades owing to their inimitable chemical, optical, catalytic, electrical, and magnetic properties. The nature of such MNPs are highly tunable, and their magnetic properties can be changed by altering their size, composition, structure, and shape [1]. Thus, these interesting features qualify MNPs to be used in a variety of environmental, industrial, technological, and medical applications. Among them, medical purposes like magnetic resonance imaging (MRI), microwave absorbers, hyperthermia, and drug supply agents are most important. Magnetic hyperthermia is the phenomenon of elevating local body temperature using MNPs to kill cancerous tumor cells [2,3]. In the nanomedical field, magnetic hyperthermia has become an important therapy owing to its antitumor activity and safety. This method also provides a choice of targeted tumor and deep tissue penetration options; however, for effective therapy and its potential as a substitute option for cancer treatment, many problems must be overcome [3]. Currently, experimental indications show that MNPs harvest heat at the nano-scale without any macroscopic temperature rises [4]. This idea comes from the fact that cancer or tumor cells are much more susceptible to heat in comparison with healthy cells. MNPs, once exposed to varying magnetic fields, absorb energy from the field and convert it into heat. This phenomenon can be explained by magnetic loss mechanisms, such as magnetic hysteresis and Néel/Brownian relaxational losses [2,4], which are particularly prominent in nanoparticles (NPs). The physiochemical characterizations of MNPs play an important role in the biomedical field, especially in understanding mechanisms associated with aggregation and agglomeration, which can be influenced by many factors, i.e., shape, dimensions, size (including grain size), etc. [5,6]. Ensuring better control over the tendency of MNPs to agglomerate is essential, especially when they are introduced into the body where they might aggregate in the bloodstream due to their high surface energy. To counteract such agglomeration, it is advantageous to modify the surface of MNPs with biocompatible, non-toxic, monodispersed, and hydrophilic polymers [7,8]. Consequently, it draws attention to formulate the MNPs by using chitosan, silica, dextran, liposomes, or cyclodextrins as a coating layer [9,10,11,12,13]. MNPs formulated in such a way have been cast nowadays as probable nanocarriers in drug transport [14,15]. The covering can shield the MNPs from oxidation and reduction that increase or decrease the magnetic properties contingent to the interface between the nanoparticle surface and ligands [16,17,18,19,20]. This mechanism is notable, especially considering the possible downsides of other MNPs that involve maghemite and magnetite that require unrestrained heating due to their high Curie temperature.

While some MNPs, such as bimetallic Ni-Cu [11], can be modified to have bio-compatibility by a silica coating, they are chemically stable and possess high saturation magnetization and a specific Curie temperature that make magnetization tunable [21,22]. The long-range order of ferromagnets has been achieved using Ni and Cu alloys [23] with the replacement of Ni by Cu. That is, although the Curie temperature of ferromagnetic material Ni is 631 K [24], it can be reduced by introducing Cu and raising its concentration to the beneficial range [25,26,27]. For instance, the initial Curie temperature 410 K of Ni_0.75_ Cu_0.25_ can be dropped to 324 K by changing the Ni ratio in the composite into 60% (Ni_0.60_ Cu_0.40_). The isolated Gd^3+^ ion is a rare earth material that contains a 4f orbit with half-filled (hereafter, we account for solitary spin involvement only for its magnetic behavior, such as in 3d metals). The magnetic moment of Gd^3+^ ion is 8.92 μ_B_ where μ_B_ is the Bohr magneton, which is very large compared to 3d transition metal ions. In addition, the magnetic order of Gd^3+^ ions, which holds even beneath room temperature, makes them assemble together [11]. The magnetic properties of Ni-Zn nano-ferrites could be improved by the replacement of a small amount of them with Gd^3+^ ions. The Gd^3+^ (0.938 Å) displays a large ionic radius compared to the transition metal ions, so the doping of Gd^3+^ ions in nanocrystalline Ni-Zn ferrites facilitates their crystal growth. The crystallite size of the composite decreases by adding the percentage of Gd^3+^ ions. The addition of Gd^3+^ ions in the composites (Ni-Zn nano-ferrites) increases their macro-strain, since the crystallite size is linked with the micro-strain, resulting in a change in microstructural properties [10]. The synthetic method of NPs strongly affects the physical and magnetic properties of them, such as particles sizes, cationic distributions, homogeneity, and dopant ions.

To minimize the risk of damaging effects of the produced heat, it is highly recommended to use a minimal number of MNPs for hyperthermia. Thus, the heating efficiency of the currently employed MNPs must be high, which makes hyperthermia a challenge to be used in practical applications [28]. Heating efficiency is usually described as a specific absorption rate or simply SAR. It corresponds to the area of the hysteresis loop for MNPs and is directly related to heat losses when these NPs are exposed to an external AC magnetic field [7]. SAR can be increased by increasing the area of the hysteresis loop. The increased area of the hysteresis loop also increases the saturation magnetization (*M_s_*) and coercive field (*H_c_*). Therefore, SAR in MNPs can also be increased by increasing *M_s_* and *H_c_* values.

Magnetic nanoparticles with a low Curie temperature, particularly for hyperthermia temperatures between 42 and 45 °C, are highly preferred along this line [10,11,14,16,29]. In the treatment of hyperthermia, the cancerous cells are killed by enriching their temperatures to the beneficial temperature ranges from 42 to 45 °C. Using this approach, the tumors are destroyed with negligible healthy tissue damage. As soon as the magnetic nanoparticles injected into oncogenic cells in this treatment are positioned in an alternating magnetic field, the heat is dissipated from nanoparticles to destroy the cancerous cells.

Magnetic materials, especially ferromagnetic ones, lose their permanent magnetic characters above a certain temperature; thus, they are unable to align in the direction of an external magnetic field, making them highly capable of producing highly localized heat from external electromagnetic energy. The loss of magnetic character occurs at the Curie temperature. That is, the Curie point sets the upper operational temperature. The lower Curie temperature of MNPs is therefore an important requirement for self-controlled hyperthermia [30]. To control the hyperthermia temperature and to attain self-regulation so that MNPs can be used as a fuse-limiter and heater, it becomes important to be able to tune the Curie temperature to a value just above the treatment temperature [31]. One such material with lower Curie temperature is doped manganese perovskite-structured La_1-x_Sr_x_MnO_3_ [32]. It is expected that most of the requirements of self-controlled hyperthermia can be fulfilled by manganite NPs with different compositions.

To improve the saturation magnetization and to tune the Curie temperature, various approaches have been developed, such as the use of different doping and coating materials or the applying of various synthesis methods. It is reported that magnetic properties can be tuned by cationic doping with different elements of varying cationic radii [33]. In this process, the lattice structure is distorted due to the doping of different cationic radii, and this distortion causes the changes in magnetic properties. For example, it is reported that a change in the doping level of Ca^2+^ and Sr^2+^ cations from x = 0.1 to x = 0.2 induces an alteration of the Curie temperature from 260 to 350 K. This tunable behavior of manganite NPs makes them suitable for biomedical applications [33].

Inspired from the above-mentioned consequence, this work is devoted to tuning the Curie temperature and enhancing the SAR of manganite NPs via the gadolinium doping enough so that they can be used in self-controlled hyperthermia applications. Core–shell gadolinium-doped lanthanum strontium manganite was fabricated using the citrate–gel auto-combustion technique, and then the nanoparticles are covered with silica shells. To investigate the samples’ characteristics, like the silica particle size and the availability of the coating, we used various techniques such as counting transmission electron microscopy (TEM), X-ray diffraction, and Fourier transform infrared spectroscopy (FTIR). NPs were heated by exerting an AC magnetic field in order to measure their Curie temperature. Dried powder samples were used to estimate the Curie temperature along with static magnetic measurements.

## 2. Results and Discussion

The lattice energy can be controlled with the aid of heat during the combustion process, which is compulsory for the development of the perovskite phase. For the atoms’ diffusion, the auto-combustion procedure might not be promising. So, the LSrp powder was taken having a crystallite size in the range of nanometers. By the annealing or heat treatment, the material starts to agglomerate and rearrange its atoms by rapid diffusion. By controlling the heat treatment time (t) and the calcination temperature (T), the nucleation stage could be controlled, leading to the development of LSrT-t powders with crystallite sizes between 9 and 57 nm.

### 2.1. XRD Study

**Figure 1:**
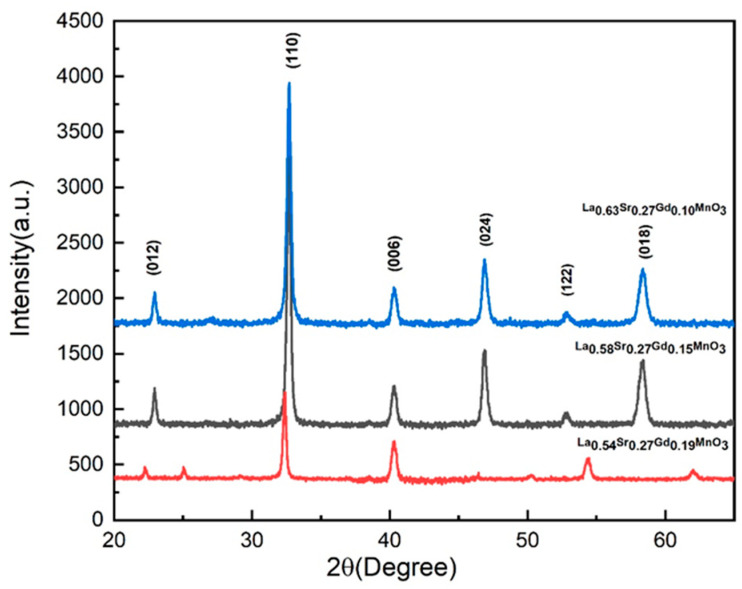
XRD graph of the La_1-x-y_Sr_x_Gd_y_MnO_3_ nanoparticles at various ratios.

XRD patterns for the annealed samples of gadolinium-doped manganite NPs can be seen in Figure 1. The XRD patterns exhibit a single-phase hexagonal perovskite structure with a space group R-3c(167)(JCPDS# 47-0444), as reported in previous studies [32]. The absence of additional peaks establishes the solubility of Gd in the manganite perovskite structure. The parent LaMnO_3_ has cubic symmetry, which transforms to hexagonal symmetry upon doping a larger ionic radius Sr^2+^ (ionic radius = 1.31 Å) in the place of smaller La^3+^ (ionic radius = 1.216 Å) and remains in the same symmetry with a low doping of Gd [1001, 1002, 1003]. The results of the elemental analysis confirm no change in elemental ratios and were matched to that of the intended synthesized ratios. The crystallite size of each sample was measured by the Sheerer formula. The crystallite size was found to be 9.25 nm, 9.7 nm, and 13.3 nm, for red, black, and blue XRD graphs, respectively. We can see that by increasing the percentage of La and decreasing the percentage of Gd, the crystallite size increases but within the nanometer range.

### 2.2. TEM Studies

**Figure 2:**
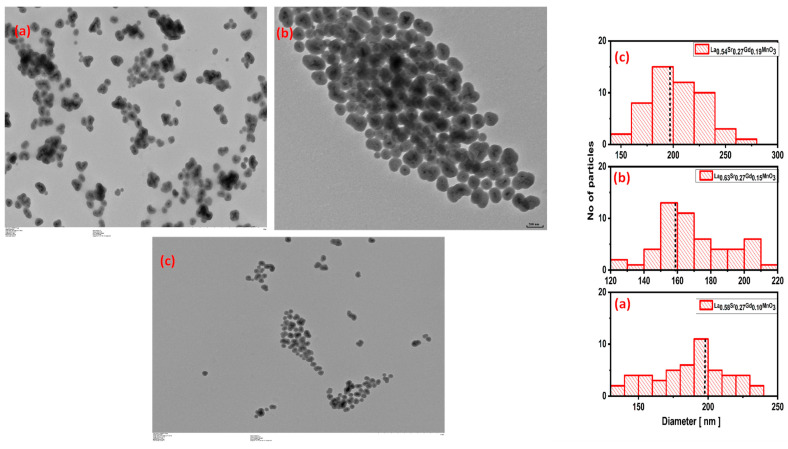
TEM images of the silica-coated La_1-x-y_Sr_x_Gd_y_MnO_3_ nanoparticles with (**a**) Gd 0.10, (**b**) Gd 0.15, and (**c**) Gd 0.19.

The observed morphology of subsequent silica-encapsulated NPs is depicted in Figure 2, where the inset shows the size distribution histogram of silica-coated NPs and their magnetic cores. One can evidently see that the NPs are successfully coated, forming stable suspension with the core size ranges that are between 160 and 180 nm. The NPs’ central layer is made of the ones that act by protecting the shell from the nearby environment and make NPs biocompatible by dropping an undesirable toxic chemical aggregation effect. See the Supplementary Materials.

### 2.3. FTIR Studies

**Figure 3:**
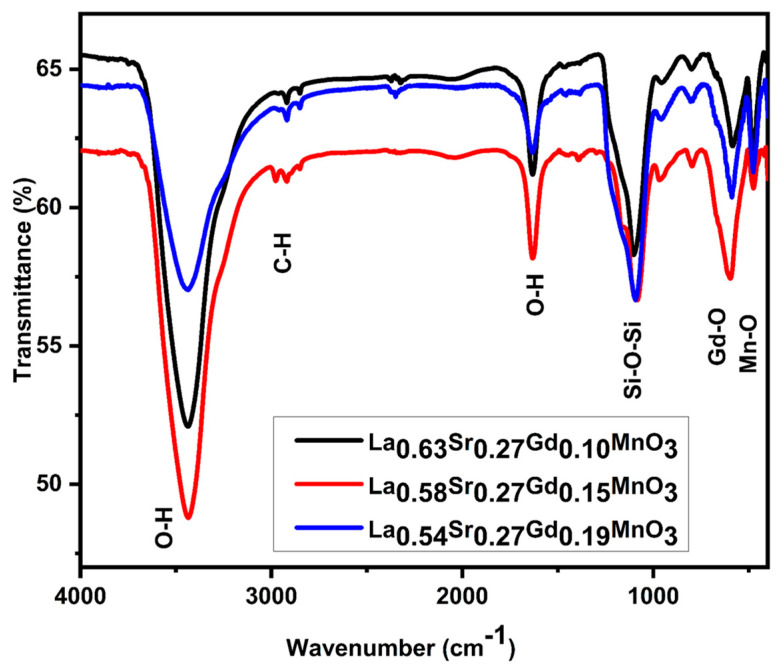
FTIR spectra for La_1-x-y_Sr_x_Gd_y_MnO_3_ nanoparticles at various ratios.

The FTIR spectrum manifests all the peaks associated with the silica and metal oxygen absorption structure, as shown in Figure 3. The absorption at nearly 600 cm^−1^ in all samples by the metal (Mn, Gd) oxygen Mn–O bond corresponds to the formation of MnO_6_ octahedra in the manganite perovskite structure [34]. The peak shifts to higher values, in turn, as 583 cm^−1^, 588 cm^−1^, and 596 cm^−1^ for x = 0.10, 0.15, and 0.19, indicating an increase in energy for the manipulation of the metal (Mn, Gd) oxygen bond. This increment of energy is caused by the increase in strain in the perovskite structure due to the inner squeeze created in the perovskite lattice with increase in dopant concentration. The maxima at 1085 cm^−1^ and 475 cm^−1^ are attributed to the antisymmetric and symmetric vibrations of Si-OH and Si-O-Si, respectively. Furthermore, the presence of a strong signal in the FTIR spectra, particularly in the range of 1600–1700 cm^−1^ due to δ (OH) groups of ethylene glycol, is indicative of absorbed water during interactions within the nanoparticles [35,36]. The peak at 2970 cm^−1^ represents the stretching mode of the asymmetric C-H bond derived from CH_2_ in tetra-orthosilicate (TEOS), and the broad band around 3500 cm^−1^ is associated with the O-H assembly, which arises from the citrate’s precursor and water molecules. See the Supplementary Materials.

### 2.4. Magnetic Measurements

**Figure 4:**
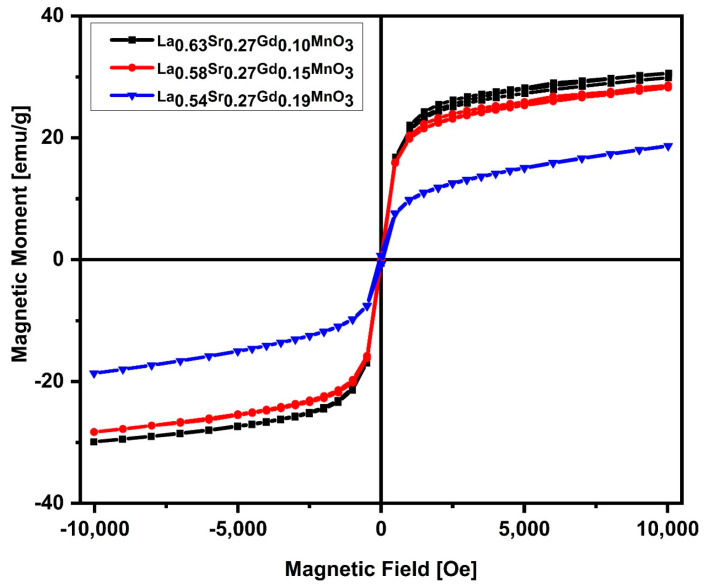
Hysteresis curve of the silica-coated La_1-x-y_Sr_x_Gd_y_MnO_3_ nanoparticles at room temperature.

Reduced magnetization values were observed for all the samples and were found to be 30, 28 and 18 emu/g for gadolinium doping of 0.10, 0.15, and 0.19, respectively, as shown in Figure 4. Manganite perovskite is a combination of three dimensional octahedra in which the B site is occupied by a transition metal ion (e.g., Mn, Fe, etc.) and resides at the center, which is surrounded by oxygen in octahedral form. Bulk LaMnO_3_ is an antiferromagnetic insulator and contains only Mn^3+^ ions. Substituting trivalent La^3+^ by divalent Sr^2+^ leads manganese to exist in both Mn^3+^ and Mn^3+^ oxidation states, inducing a double-exchange interaction, which leads to ferromagnetic ordering [21]. Incorporating gadolinium (Gd) into the crystal lattice structure causes distortions in the lattice, thereby influencing the lengths and angles of bonds. These distortions alter the magnetic interactions within the material, ultimately leading to alterations in its magnetic properties. The decrease in magnetization is closely related with the presence of disordered surface spins and lattice distortions caused by the substitution of smaller gadolinium ions within the lattice structure, which leads to deteriorated ferromagnetic interaction [22,23]. Saturation magnetization values are comparable to those reported in the literature [32]. The complete magnetic saturation was not found in the result. The reason behind unsaturated magnetization could be the breaking of a double-exchange interaction at the NP surface due to oxygen deficiencies. This breaking results in disordered spins that act para-magnetically, leading to the non-saturation of magnetization.

### 2.5. Curie Temperature Measurements

**Figure 5:**
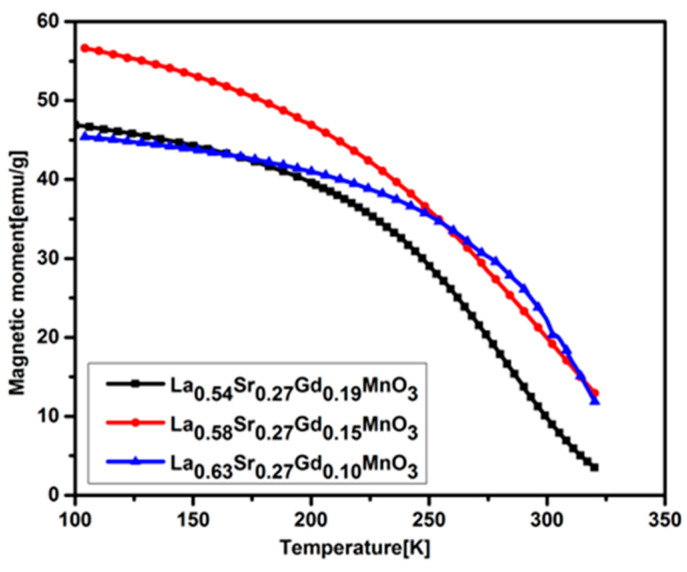
Magnetization as the function of temperature for the silica-coated La_1-x-y_Sr_x_Gd_y_MnO_3_ nanoparticles at 1000 Oe.

The Curie temperature measurements were performed, whereas all samples display soft ferromagnetic behavior by the value of coercivities residing within the range of 8–29 Oe at 200 rpm mechanical milling and 42–87 Oe at 500 rpm mechanical milling. Consequently, we obtained the crystallite size within the range of 13–43 nm by shifting the milling speed and time along with the coercivities lying among 8 and 87 Oe where the magnetization value *M* @ 15 kOe ranges from 7 to 73 emu/g. To measure the thermal demagnetization, the temperature was kept in the range of 100 to 350 K beneath the measuring magnetic field of 1000 Oe, as shown in Figure 5. The Curie temperature was assessed from the magnetization (M-T) curve obtained from the temperature range of 100 to 350 K. A trend of decreasing Curie temperature with increasing gadolinium doping was observed. One can also estimate the Curie temperature from a heating curve obtained during calorimetric measurements. Both the Curie temperature T_c_ and coercivity values are noticeably reduced after the doping of gadolinium in lanthanum strontium manganite compared to those of undoped manganite reported in the literature [32,37]. Tokura et al. demonstrated that LaMnO_3_ transforms to a ferromagnetic state from antiferromagnetic with strontium concentration between 0.17 and 0.45 and depicted the Curie temperature approximately based on the magnetic phase diagram [38]. The change in transition temperature in La_1-y_Sr_0.27_Gd_y_MnO_3_ compared to La_0.63_Sr_0.27_MnO_3_ [32] could be accredited due to the cation size and the lower Curie temperature of gadolinium. A double-exchange interaction is responsible for the magnetic properties of doped manganites [39]. Two factors are detrimental for the strength of the double-exchange interaction mechanism: the manganese oxidation state and the angle between the manganese cations and oxygen anions. The double-exchange interaction, in particular, is prominent when there is a maximal overlap of d- and p-orbitals in Mn^3+^-O_2_-Mn^4+^ [39].

A great variation of T, from −13 to 77 °C, was experimentally found when the dopant cationic radii are bigger than La^3+^ [40]. However, with smaller dopant cation radii, the Curie temperature decreased due to the distortion in lattice structure in the form of bond angles and lengths, as reported in previous studies [40].

Gadolinium cationic radii are smaller than lanthanum; therefore, the substitution of gadolinium will result in the creation of distortion and defects in the crystal lattice structure. The consequence of this distortion in the perovskite unit cell causes a variation in bond lengths and angles, which in turn leads to tuning of the Curie temperature and magnetic properties. The lower Curie temperature of gadolinium could be another cause for the decrease in the Curie temperature in gadolinium-doped manganite. One can observe from the M-T curve that magnetization is increasing with cooling.

### 2.6. Magnetic Heating

**Figure 6:**
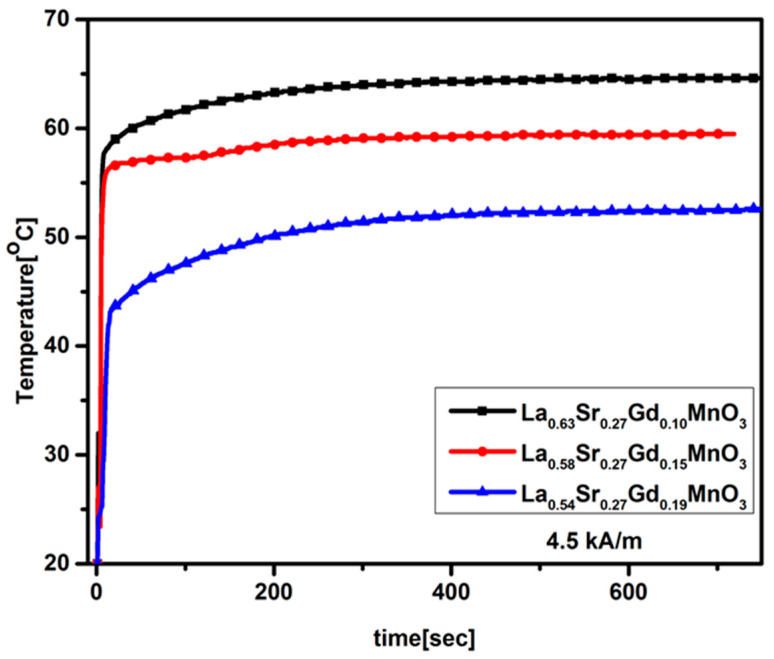
Variation of temperature versus time curve for powder samples.

**Figure 7:**
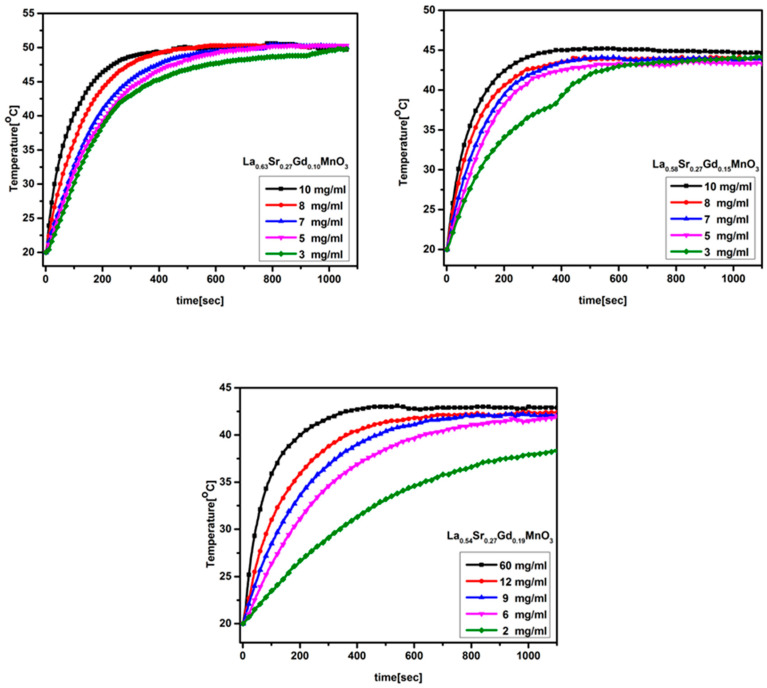
Variation of temperature over time for all three samples.

Magnetic heating experiments were performed for dry powder and a colloidal form of silica-coated NPs in water under an AC field of amplitude 4.4 kA/m and frequency of 216 kHz. For powder samples with no water, as shown in Figure 6, the temperature quickly reaches saturation and then stabilizes. One can deduce the transition temperature from the powder sample temperature vs. time graph by comparing it with that of the magnetization vs. temperature data. The temperature vs. time graph for the samples recorded during the application of an AC magnetic field is shown in Figure 7. For colloidal Si/MNPs with x = 0.15, the temperature stabilizes close to the Curie temperature after the initial rise, matching the therapeutic temperature range of self-controlled hyperthermia. Additionally, the presence of a silica coating has an impact on the transfer of heat to the nearby environment. When the stabilization temperature is achieved, the overall heat loss is in equilibrium with the hyperthermic heat production. It is worth noting that the saturation temperature in a colloidal suspension tends to be lower than that in a powder sample, which is primarily because there is minimal heat loss to the environment in the case of the powder sample.

The specific absorption rate for NPs dispersed in water was calculated using the equation:

(1) $$SAR\;=\;\sum \;C_{p}\frac{1}{m_{Mn+Gd}}\frac{dT}{dt}$$

expressed in units of W/g. Here, *C_p_* is the specific heat capacity of the aqueous suspension of the NPs, which is roughly equal to specific heat capacity of water, i.e., 4.18 Jg^−1^K^−1^. *dT*/*dt* is the slope of the initial heating curve, and *m_Mn+Gd_* is the mass of the magnetic components in the sample. The specific absorption rate was calculated for core–shell silica magnetic core from the temperature–time curve according to the above equation, and it was found to be 73 W/g, 108 W/g, and 136 W/g based on the combined weight of manganese and gadolinium content in 10 mg/mL, 10 mg/mL, and 12 mg/mL samples of Gd0.19, Gd0.15, and Gd0.10 NPs, respectively. The obtained values are comparably higher than previous reported values [35]. The incorporation of gadolinium enhances magnetic heating while reducing the Curie temperature.

**Figure 8:**
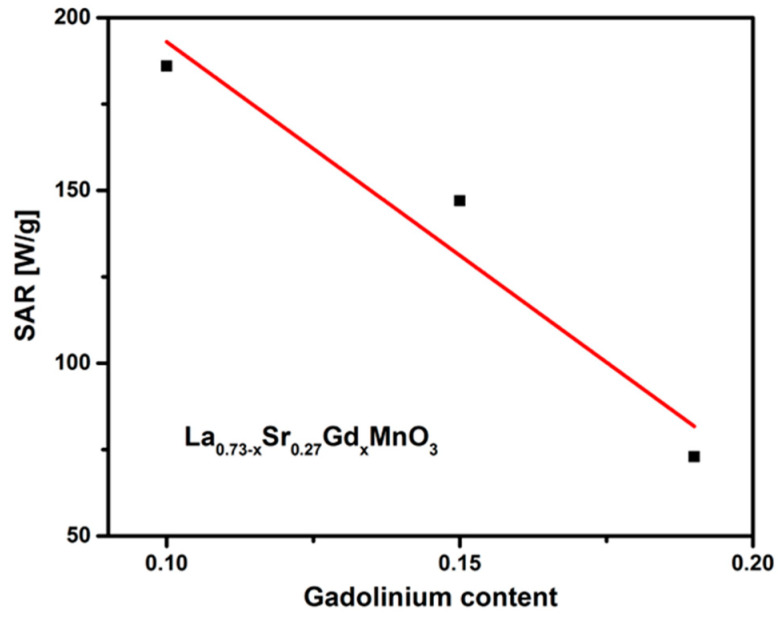
Variation of SAR with gadolinium content.

Variation in the SAR value with the gadolinium content is shown in Figure 8, which exhibits a trend of decreasing SAR with the increase in the gadolinium content. For La_0.58_Sr_0.27_Gd_0.15_MnO_3_, although the SAR value is not the largest of the three samples, the stabilization temperature is below the Curie temperature. NPs can only sustain their magnetization, and hence can generate required heat under the application of a magnetic field, when the hyperthermia temperature is kept below the Curie temperature. Hence, we can conclude that La_0.58_Sr_0.27_Gd_0.15_MnO_3_ NPs meet the hyperthermia requirement with a self-regulated temperature of about 44.5 °C.

## 3. Materials and Methods

### 3.1. Material Synthesis

The synthesis process of silica-encapsulated manganite NPs involves two steps. Firstly, the gadolinium-doped La_1-x-y_Sr_x_Gd_y_MnO_3_ (y = 0.10, 0.15, 0.19) was fabricated using a citrate gel auto-combustion technique based on the previous report [41]. In a typical synthesis, the analytical grades of La(NO_3_)_3_·6H_2_O, Sr(NO_3_)_2_, Gd(NO_3_)_3_, and Mn(NO_3_)_2_ in stoichiometric quantities were dissolved in 60 mL of distilled water for intended compositions trailed by the addition of ammonium hydroxide NH_4_OH (25 vol%, Sigma Aldrich, Seoul, Republic of Korea) to peptize the solution into a stable emulsion. For the fuel and gel-forming agent, the citric acid and ethylene glycol were added to the solution. The solution stirred for 30 min at room temperature, and then the mixture was kept at 120 °C until polymer gel formation was achieved by a polyesterification reaction. Through this, dry powder was obtained at 160 °C after some time. The black fluffy powder was formed spontaneously after self-ignition of the yellowish dry powder in a microwave oven. Calcination was carried out at 450 °C to obtain the manganite phase, after which we removed the residual impurities for the completion of the reaction. The final composite was annealed at 850 °C for 3 h to improve crystallinity and was used for silica coating and further characterization.

### 3.2. Silica Coating

Silica encapsulation was carried out using the method reported by Kaman et al. [35]. First, 100 mg of the obtained NPs was dispersed in water and subjected to an ultrasonic probe for 10 min to ensure a good dispersion of NPs. Subsequently, particles were dispersed in water using a few drops of nitric acid in ultrasonic bath, which was followed by centrifugation and redispersion in 1 M citric acid. Finally, the surface-charged NPs were collected using a centrifuge, alkalized by a few drops of ammonia, and then added dropwise to a mixture of ethanol (150 mL), water (35 mL), and ammonia (10 mL). The solution was mechanically stirred at 40 °C for 4 h after the addition of TEOS. The magnetic separation process was used to separate the final product of silica-coated NPs from solution and washed numerous times in water and ethanol, which was followed by dispersion in water for further characterization.

### 3.3. Characterization

XRD analysis was performed for the crystallographic structure analysis and phase cataloging using an X’pert PRO PAN analytical diffractometer containing Cu-K*_α_* radiation of wavelength λ = 1.5406 Å. The angle 2θ was varied from 20 to 70 degrees, and the scan rate was 2 degree/min. FTIR was employed to study the various vibrational modes and analyze the metal organic interlinkage. The NPs’ morphology and size distribution were observed using TEM (H-7600, Hitachi Ltd. Tokyo, Japan). The magnetic phase was investigated by temperature-dependent magnetization in the range of 100 to 330 K using a vibrating sample magnetometer (LDJ-9500).

The Induction heating system (OSH-120B, OSUNG HITECH, Seoul, Republic of Korea) was used to study the magnetic heating response. The NPs in the form of aqueous and powder samples are placed in an RF coil with a fixed frequency of 216 kHz and an AC field of amplitude 4.4 kA/m. To measure the temperature of the samples, an infrared (IR) thermometer was used.

## 4. Conclusions

Silica-encapsulated-gadolinium doped lanthanum strontium manganite NPs with different gadolinium concentrations were effectively synthesized using the citrate–gel auto-combustion method, which was followed by coating with silica with tunable Curie temperatures and enhanced SAR. From the results of our study, we demonstrated that the lanthanum strontium manganite NPs’ Curie temperatures are reduced when gadolinium was introduced as a dopant. This essential factor guarantees that the NPs can maintain their magnetization and can produce heat efficiently in response to the applied external magnetic field, aligning with the hyperthermia temperature range typically used for cancer therapy. The production of core–shell NPs, with magnetic core diameters ranging from 20 to 50 nm, has been confirmed by structural investigation. The NPs were effectively shielded by the silica coating, making them biocompatible and reducing any potential harmful effects from chemical aggregation. Additionally, it was found that gadolinium significantly modified the magnetic characteristics of the NPs, producing lattice distortion effects to lower magnetism. Our magnetic heating experiments demonstrated that the NPs could achieve a self-regulated temperature close to the desired therapeutic range for hyperthermia. This result indicates a significant advancement that reduces the risk of overheating and potential damage to healthy tissues during cancer treatment.

## Data Availability

Data are contained within the article and Supplementary Materials.

## Supplementary Materials

The following supporting information can be downloaded at https://www.mdpi.com/article/10.3390/molecules28237860/s1.
