# Supplementary material for: Improvement of the Self-Controlled Hyperthermia Applications by Varying Gadolinium Doping in Lanthanum Strontium Manganite Nanoparticles
Source: Molecules. 2023 Nov 30;28(23):7860. doi: 10.3390/molecules28237860 (PMC10707745; doi:10.3390/molecules28237860)
Supplement: Supplementary file 1 [file molecules-28-07860-s001.zip › molecules-2700432-supplementary.pdf]

Supplementary Materials

Improvement of the Self-Controlled Hyperthermia Applications by Varying Gadolinium Doping in Lanthanum Strontium Manganite Nanoparticles

Ashfaq Ahmad [1,†], Hassan Akbar [2,3,†], Imran Zada [1], Faiza Anjum [4], Amir Muhammad Afzal [5], Subhan Javed [4], Muhammad Muneeb [4,] Asghar Ali [4,*] and Jeong Ryeol Choi [6,*]

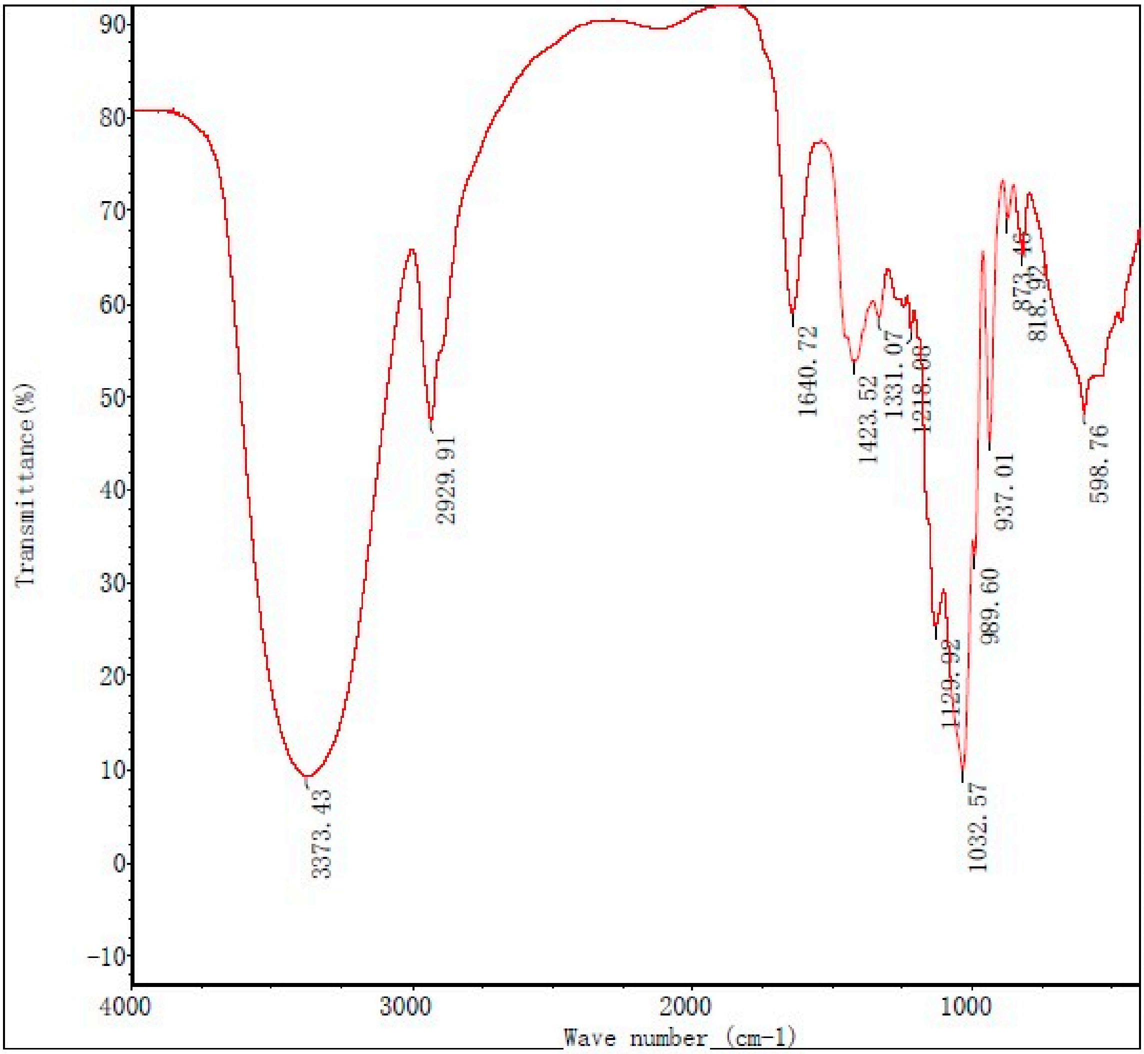

Infrared spectrum results

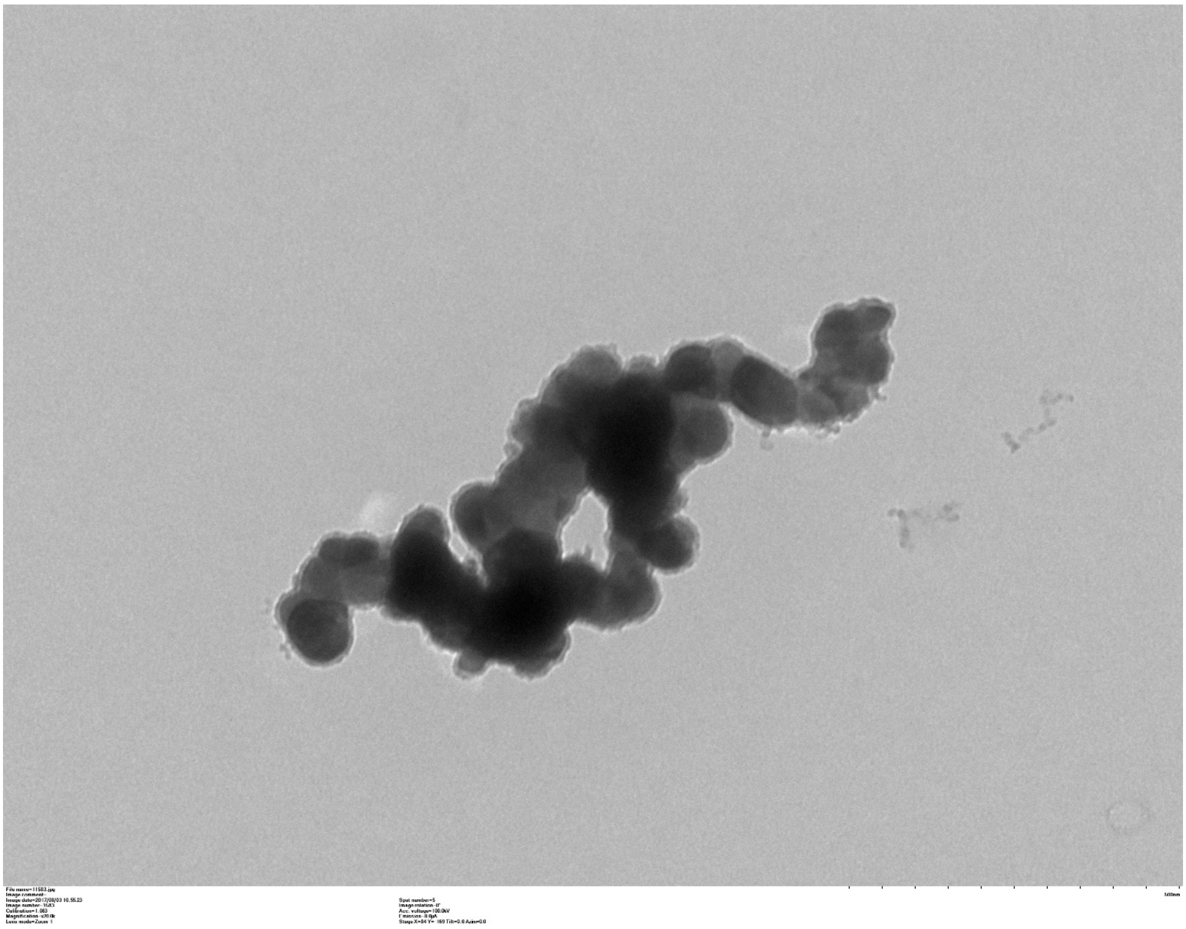
File name=11583.jpg
Image date=2017/08/03 10.55.23
Calibration=1.083
Lens mode=Zoom-1
Spot number=5
Acc. voltage=100.0kV
Stage X=84 Y=-169 Tilt=0.0 Azim=0.0
500nm

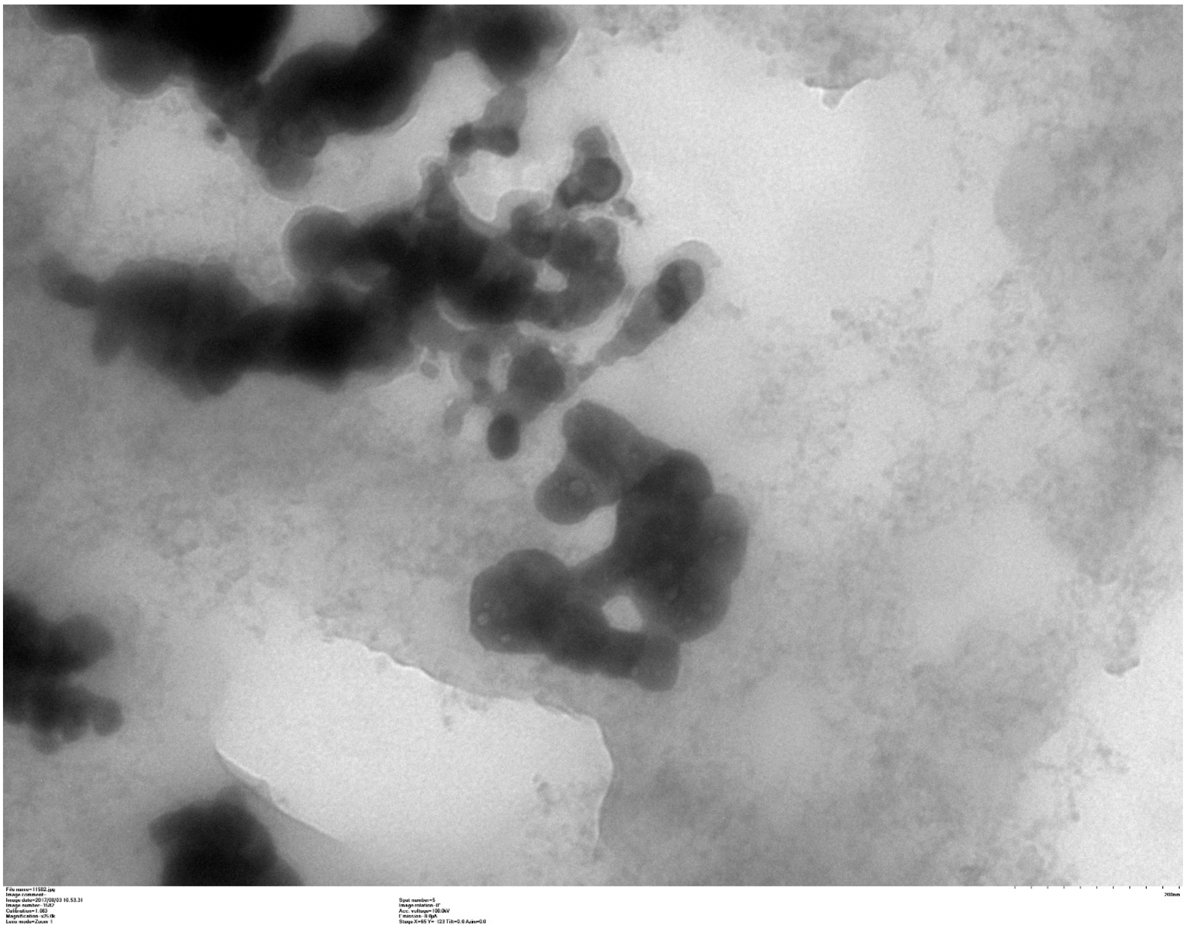

TEM images of the $La_{1-x-y}Sr_xGd_yMnO_3$ nanoparticles

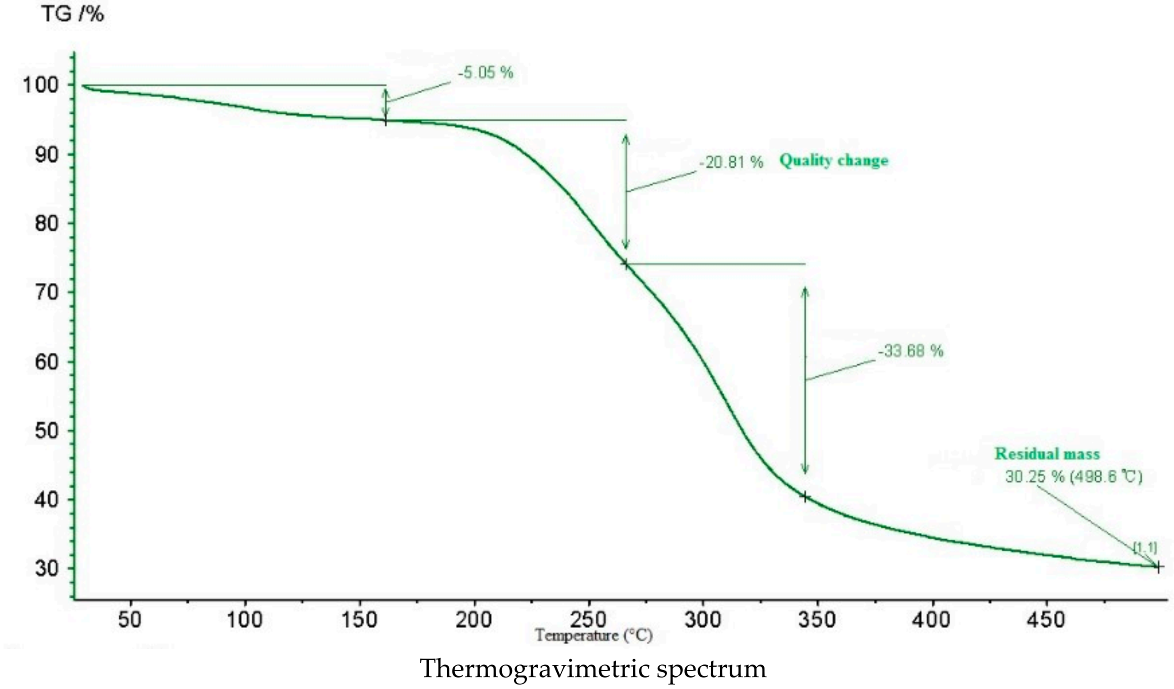

Thermogravimetric spectrum
